# Gene network analyses unveil possible molecular basis underlying drug-induced glaucoma

**DOI:** 10.1186/s12920-021-00960-9

**Published:** 2021-04-19

**Authors:** Ruo-Fan Ding, Qian Yu, Ke Liu, Juan Du, Hua-Jun Yin, Zhi-Liang Ji

**Affiliations:** State Key Laboratory of Stress Cell Biology, School of Life Sciences, Xiamen University, Xiamen, 361102 Fujian People’s Republic of China

**Keywords:** Drug-induced glaucoma, Adverse drug reaction, The weighted gene co-expression network analysis, Glaucoma biomarkers

## Abstract

**Background:**

Drug-induced glaucoma (DIG) is a kind of serious adverse drug reaction that can cause irreversible blindness. Up-to-date, the molecular mechanism of DIG largely remains unclear yet due to the medical complexity of glaucoma onset.

**Methods:**

In this study, we conducted data mining of tremendous historical adverse drug events and genome-wide drug-regulated gene signatures to identify glaucoma-associated drugs. Upon these drugs, we carried out serial network analyses, including the weighted gene co-expression network analysis (WGCNA), to illustrate the gene interaction network underlying DIG. Furthermore, we applied pathogenic risk assessment to discover potential biomarker genes for DIG.

**Results:**

As the results, we discovered 13 highly glaucoma-associated drugs, a glaucoma-related gene network, and 55 glaucoma-susceptible genes. These genes likely played central roles in triggering DIGs via an integrative mechanism of phototransduction dysfunction, intracellular calcium homeostasis disruption, and retinal ganglion cell death. Further pathogenic risk analysis manifested that a panel of nine genes, particularly *OTOF* gene, could serve as potential biomarkers for early-onset DIG prognosis.

**Conclusions:**

This study elucidates the possible molecular basis underlying DIGs systematically for the first time. It also provides prognosis clues for early-onset glaucoma and thus assists in designing better therapeutic regimens.

**Supplementary Information:**

The online version contains supplementary material available at 10.1186/s12920-021-00960-9.

## Background

Glaucoma is a severe disease that affects more than 70 million people worldwide. As a leading cause of irreversible blindness, glaucoma’s clinical manifestations include optic disc atrophy, visual field defect, and visual acuity loss; it brings great inconvenience and desperate darkness to the patients [1]. Drug-induced glaucoma (DIG) is a special kind of glaucoma, which may be caused by many commonly used drugs, such as dexamethasone, paclitaxel, fluoxetine, and perphenazine in normal chemotherapy [2, 3]. Generally, the DIGs can be classified into two types: the open-angle glaucoma (OAG) with elevated intraocular pressure (IOP) which is mainly caused by the steroids, such as betamethasone, prednisolone, and dexamethasone; and the closed-angle glaucoma (CAG) with blocked trabecular network outflow path which is mainly caused by the non-steroidal drugs such as anticholinergic drugs and epinephrine drugs. Some antitumor drugs, such as docetaxel and paclitaxel, can also induce OAG via unclear internal mechanisms [2–4]. As of December 2019, a total of 13,961 cases (including 12,195 serious cases) of DIGs have been reported in the FDA Adverse Event Reporting System (FAERS) of the United States.

In recent years, extensive attention has been paid to discovering the molecular mechanisms beneath glaucoma. Both the animal model and the Genome-Wide Association Studies (GWAS) were adopted to explore susceptible pathways or genes beneath glaucoma and obtained some encouraging achievements. For instance, Weinreb linked glaucoma with multiple pathways of lipid metabolism, inflammation, autoimmunity, and oxidative stress [1]. Razeghinejad attributed forward movement of the iris–lens diaphragm to the closed-angle glaucoma [3]. Clark proposed glucocorticoid-induced OAGs likely led by inhibition of matrix metalloproteinases [5]. Danford suggested the primary OAGs related to *MYOC*,* OPTN*,* CAV1*, and *CAV2* [6]. Regretfully, these efforts failed to identify a strong genetic factor in glaucoma development. The surgery-simulated glaucoma in the animal model experiment is to some extent different from the real clinical case, which may bring unpredictable errors. Taking the advantages of high-throughput technology, the GWAS studies discovered a list of genes and genomic mutations susceptible to DIG on a large scale [7]. We summarized these efforts in Additional file 1: Table S1, in which information of four potential DIG-associated genes (*MYOC*, *GPNMB*, *HCG22*, and *TSPO*) were given in brief. Regretfully, the GWAS applications are largely limited by sample size and financial budgets. Moreover, many of the susceptible genes identified by the GWAS studies have relatively small penetration to glaucoma because of the inherent limitations of GWAS itself, for instance, the inability to fully explain the genetic risk of common diseases and the difficulty in figuring out the true causal associations [8]. Therefore, more susceptible genes are awaiting discovery from the larger DIG cohorts, and at the same time, well-defined phenotypes are expected to delineate the complex genetic architecture of glaucoma [9].

In the present study, we attempt to integrate and analyze the heterogeneous data of clinical records and transcriptomes to identify the drugs that are highly associated the DIGs, unveil the gene interactions underlying glaucoma development, extract the pivot pathways and genes underlying glaucoma, and eventually discover the potential gene biomarkers for DIG prognosis.

## Methods

### Data source and preprocessing

#### The drug-ADR relations

The drug-ADR relations were derived from the Adverse Drug Reaction Classification System (ADReCSv2.0) [10]. Overall, 173 distinct glaucoma-inducing drugs were extracted from the ADReCS, as well as their comprehensive drug information and ADR information. The drug indication information was subject to the Anatomical Therapeutic Chemical Classification System (ATC Code).

#### The drug-treated gene expression profiles

The drug-treated gene expression profiles were downloaded from the LINCS project [11]. Overall, 1,319,138 gene expression profiles were obtained from the LINCS database, covering 25,149 chemicals, 12,328 genes, 76 cell types, and 5,420 combinational conditions (cell line, drug dosage, and exposure time). For every drug-treated profile, the gene expression perturbation to the control was determined by the LINCS L1000 project using the Characteristic Directions (CD) geometrical approach [12] and as well the significance (*p* value) of perturbation was calculated [11]. In addition, the top perturbed genes were elected as the differentially expressed genes (DEGs). The profiles were then preprocessed as following: (1) excluded the experiments without biological replicates; (2) for every drugs, chose the mostly perturbed profiles (with minimum *p* value and *p* < 0.01) as the representatives. After the data preprocessing, overall 1114 gene expression profiles were retained for later analysis, covering 299 chemicals (272 approved, 4 experimental, and investigational drugs), 12,328 genes, 9 cell types, and 27 combinational experiment conditions (drug dosage and exposure time) (Additional file 2: Table S2).

### Mining drug-glaucoma association

To obtain reliable drug-glaucoma associations, we downloaded 9,772,360 adverse drug event (ADE) reports, dating from Jan 2004 to Dec 2018, from the FDA Adverse Event Reporting System (FAERS) of the United States. Excluding the reports from unreliable sources and non-single active ingredient drugs, a total of 2,069,653 reports were used for association analysis, including 1095 distinct single-ingredient drugs and 10,287 adverse reactions. The ADR terms were standardized by referring to the ADReCS. Subsequently, we calculated the odds ratio (OR) for every drug-ADR pairs following the standard procedure. This analysis involved 106 distinct drug-glaucoma associations after ADR standardization.

### Tissue propensity analysis

We manually mapped the nine studying cell types against the System Organ Class (SOC) of the ATC system, each cell type corresponded to only one organ. As the result, four organs were involved (Additional file 3: Table S3). Of these organs, the ATC code of a drug at the SOC level were taken as its expected site-of-action (SOA). For every drugs involved in this analysis, we calculated the average expression changes of its DEGs in response to the drug treatment. The average perturbations (the average expression changes of DEGs) of drugs in SOA and non-SOA groups in each cell type were statistically analyzed.

### The weighted gene co-expression network analysis (WGCNA)

The weighted gene co-expression network analysis (WGCNA) was conducted on 13 selected drug-treated gene expression profiles using the R package “WGCNA” [13], and the soft threshold for network construction was set to 5 (with scale-free coefficient R^2^ = 0.88). Upon the gene network, the eigengene dendrogram was constructed using the average linkage hierarchical clustering and the dynamic tree-cutting algorithm [14]. A dissimilarity threshold of 0.3 (or similarity threshold of 0.7) was set for the eigengene dendrogram to reduce network complexity by merging similar modules. The genes in the same module were taken as the co-expressed genes. For each gene module, the module eigengenes (MEs) were the first principal component of the expression matrix.

### Identification of glaucoma-associated genes

For the selected gene module, we quantitatively measured the module membership (MM) between MEs by the Pearson Correlation analysis. The highly connected genes (MM > 0.95) were selected as core genes for subsequent protein–protein interaction (PPI) analysis. The PPI data for highly connected genes were obtained from the STRING database (v10.5, species: Homo sapiens) by satisfying PPI enrichment significance *p* < 0.01 [15]. Upon the PPI data, we re-constructed the PPI network using the Cytoscape software (version: 3.7.1) [16], from which we extracted the hub genes (Maximal Clique Centrality ≥ 5 and Betweenness centrality > 0) as the potential glaucoma-associated genes via the CytoHubba plugin.

### Functional enrichment analysis

Gene Ontology (GO) and KEGG pathways enrichment analysis were conducted on the genes of interest using the R package ClusterProfiler [17]. Fisher’s exact test was used to evaluate the significance of the biology processes or pathways. The false discovery rate (FDR) correction method (Benjamini and Hochberg) was used to evaluate the multiple tests in the enrichment analysis.

### The permutation test

We performed the permutation test to measure the significance of gene expression differences under the treatment of 13 glaucoma-associated drugs and 63 non-glaucoma-inducing drugs. The drugs of both drug groups all belonged to the ATC category "S" (Sensory organs). For each of the studying genes, the permutation test was demonstrated independently on a null hypothesis (i.e. no gene expression difference between two drug groups). We simulated the drug group composition by resampling the group members 10,000 times randomly, calculated the gene expression difference between drug groups, and determined the distribution of gene expression difference. At the same time, we calculated the real gene expression difference between two drug groups for each gene. Subsequently, we located the real difference at the random expression difference distribution, upon which the significance *p* value was determined by counting the proportion of samples less than the real difference value. A *p* < 0.05 will reject the null hypothesis, indicating there exists a significant gene expression difference between the two drug groups.

### Pathogenic risk assessment

The pathogenic risk assessment was demonstrated in this study by calculating the odds ratio (OR) value of the selected gene to glaucoma. The odds ratio analysis took the 77 gene expression profiles as the independent variables *x* and the binary consequence of glaucoma (1 for glaucoma and 0 for non-glaucoma) as the response variable *Y* [12]. We assumed there existed a linear relationship between the independent variables *x* and the log-odds of ${\text{Y}} = 1$, denoted as $p = {\text{P}}\left( {{\text{Y}} = 1} \right)$. This linear relationship can be determined by:

1 $$\ell = \log_{e} \frac{p}{1 - p} =\upbeta _{0} +\upbeta _{i} x_{i}$$

where *ℓ* stands for the log-odds, ${\upbeta }_{i}$ stands for the parameter of the Logistic Regression model. According to the relationship of the response variable *Y* and independent variable *x*, we drew the receiver operating characteristic curve (ROC curve) and calculated the area under the curve (AUC) for each variable *x*. In this study, the logistic regression was performed using the R function glm (R version v3.60), and the AUC was calculated by R function colAUC from caTools (v1.18.0).

## Results

### Identify the glaucoma-associated drugs

**Fig. 1:**
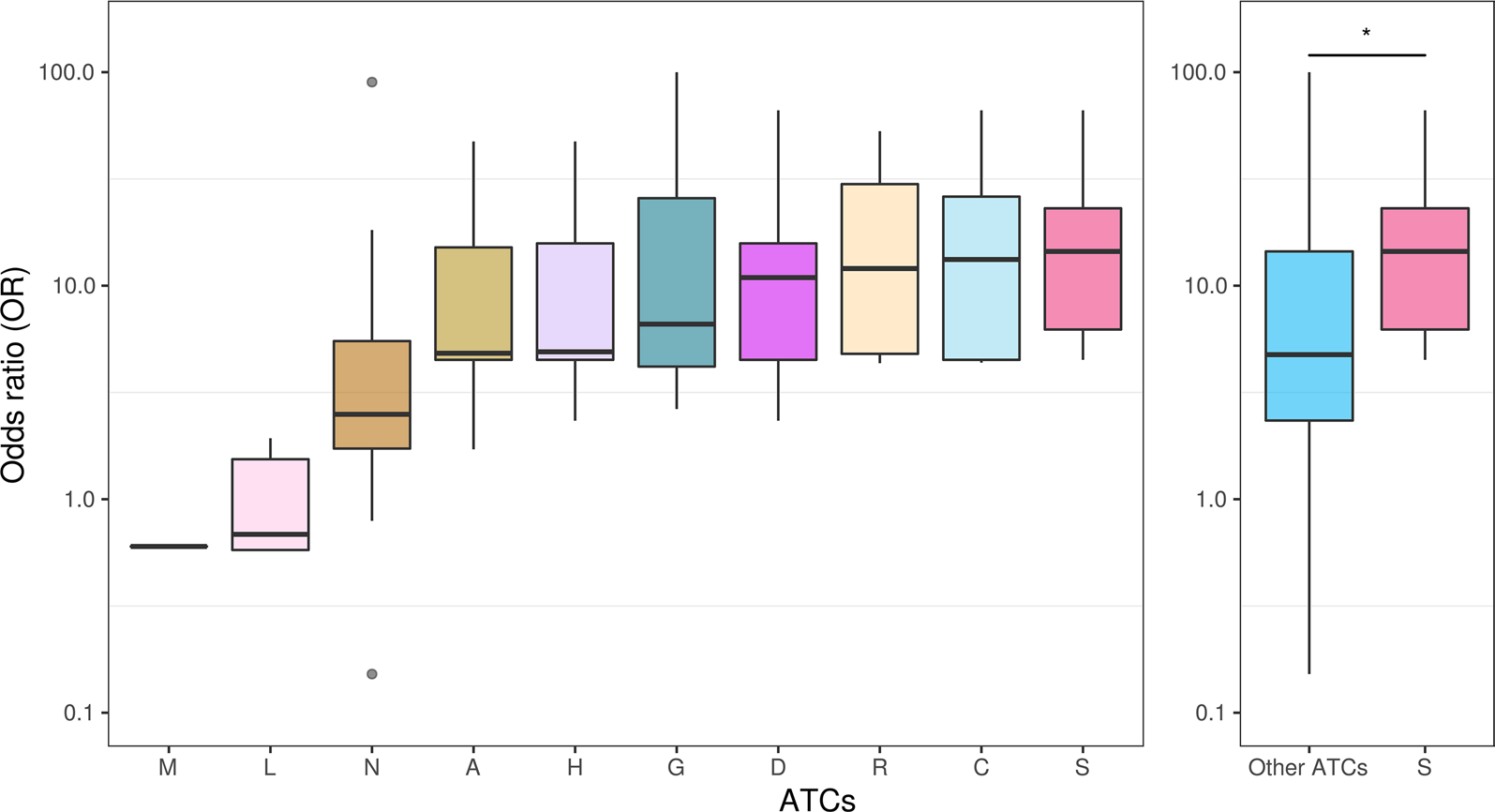
Identification of significant drug-glaucoma associations. **a** The association strength (odds ratio) of 71 glaucoma-inducing drugs among 10 ATC categories. **b** The association strength comparison of 10 drugs in ATC category “S” (Sensory organ) and 61 drugs in other categories. The drug-glaucoma association strength were determined upon 2,069,653 historical ADEs of the FARES database using the odds ratio analysis. One-sided Wilcox test was used to determine the significance of the difference between these two groups. The star symbol (*) stands for *p* value < 0.05. The drugs of category S had higher association strength than that of other drug categories

Overall, 173 glaucoma-inducing drugs were derived from the ADReCSv2.0 database. These drugs can induce glaucoma at different occurrences in patients as estimated in the drug label. To determine the drug-glaucoma associations quantitatively, we analyzed 9,772,360 real-world historical ADEs of the FAERS using the odds ratio analysis and the Fisher test. A list of 25 significant (OR > 2 and *p* < 0.05) drug-glaucoma associations were summarized in Additional file 4: Table S4. In the list, 13 drugs exhibited strong associations with glaucoma (ORs > 10 and *p* < 0.01). Additional analysis found that drugs of the ATC code "S" (Sensory organs) category were more significantly associated with glaucoma compared to other non-S category drugs (*p* < 0.02) (Fig. 1). The average ORs for S category drugs and the non-S category drugs were 21.13 and 14.74, respectively.

**Fig. 2:**
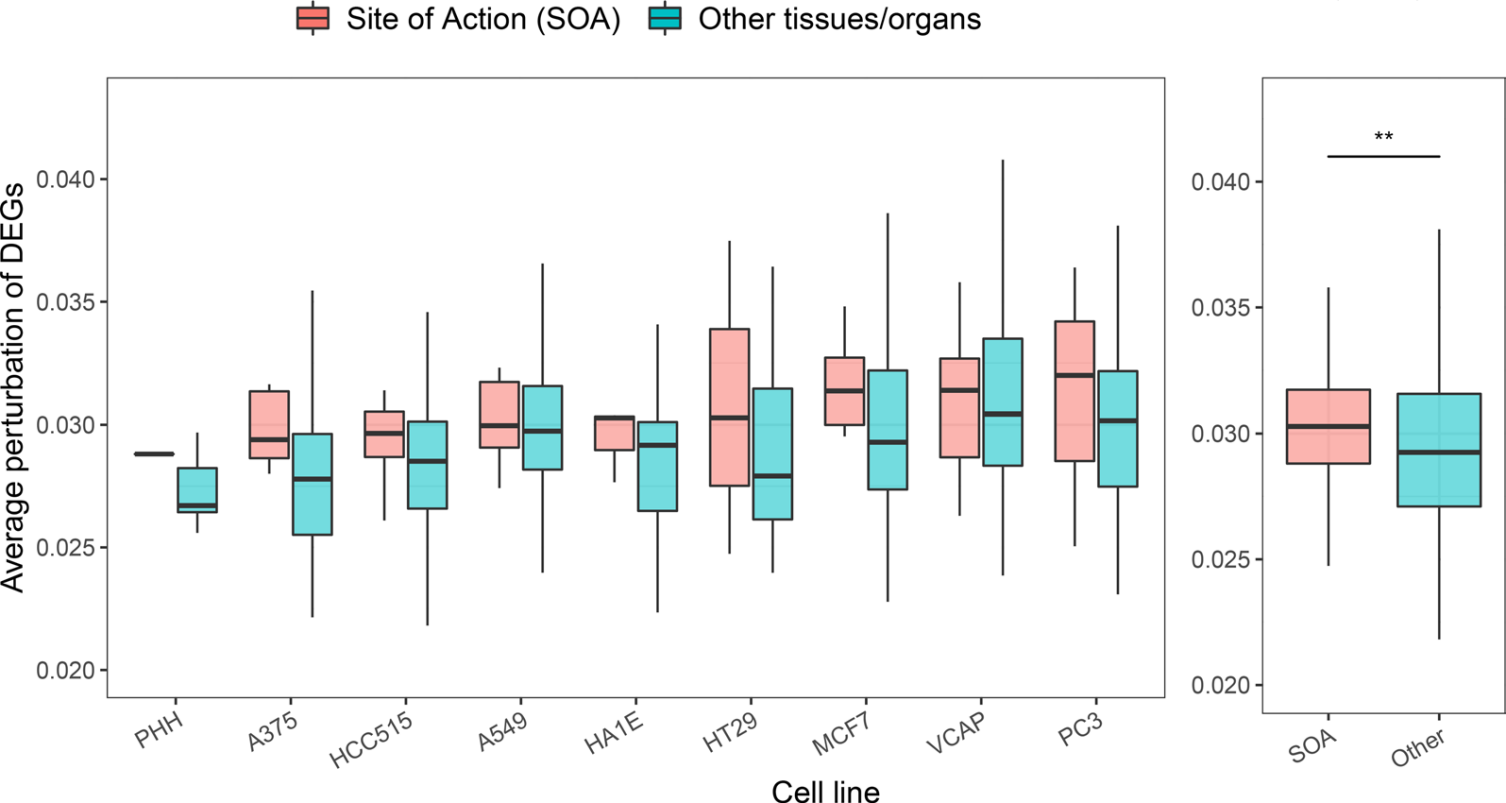
The tissue propensity of drug-induced glaucoma. The X-axis stands for the nine different cell types and Y-axis stands for average perturbation of the DEGs. The pink box stands for the expected Site-of-Action (SOA) of the drugs. The blue box stands for the tissues/organs other than the SOA of the drugs. One-sided Wilcox test was used to determine the significance of the difference between SOAs and other tissues/organs. The star symbol (*) stands for *p* < 0.05. The results manifested that the drugs intended to interfere gene expression mostly at its SOA

Furthermore, we conducted a comparative analysis of genome-wide gene expression changes over different cell lines in response to drug treatment. The result manifested that the gene expressions were more perturbed in the constituted cells of the site-of-actions (SOAs) of drugs than in the cell lines of other tissues/organs (Fig. 2). This suggested that many high occurrence ADRs were likely caused by the augment effects of gene expression perturbation. Therefore, we speculated that DIGs might tend to occur on the sensory organs, particularly the eyes. Accordingly, 13 drugs were selected for later mechanistic analyses by satisfying both criteria of belonging to the S category and having a strong association with glaucoma (Additional file 5: Table S5).

Before moving forward, we made a structural similarity measurement of the selected 13 glaucoma-associated drugs using the Tanimoto coefficient, in which the chemical structures were represented by the binary fingerprint value. The measurement revealed a low similarity between drugs (the average Tanimoto Coefficient = 0.43). As well, we evaluated the dispersion degree of physicochemical properties for the 13 drugs using the coefficient of variation (CV). The results also supported the high variability of drugs (average CV = 0.358). Both analyses manifested that the 13 glaucoma-associated drugs were highly diverse in structure and physicochemical properties; and as a consequence, it is unlikely for the drugs to induce glaucoma in a simple way of targeting the same protein.

### Unveil the gene interactions underlying the glaucoma development

To unveil gene interactions underlying the DIGs, we carried out WGCNA analyses on gene expression profiles under the treatment of the 13 selected glaucoma-associated drugs as described in “Methods” section. The analysis extracted 72 gene modules (subnetworks) from the whole gene co-expression network via average linkage hierarchical clustering and dynamic tree-cutting algorithm. To reduce the network complexity, we set a dissimilarity threshold of 0.3 (or similarity value = 0.7) to merge gene modules in the dendrogram of module eigengenes (MEs) and eventually obtained 27 modules (Additional file 7: Figure S1). These 27 gene modules or subnetworks represented the functional gene groups in response to the treatment of 13 glaucoma-associated drugs. Subsequent function enrichment analyses on these 27 gene modules identified one module (involving 5,968 genes) potentially linked with DIG. This gene module contained the biological function terms related to sensory organ such as "visual perception" (GO:0007601), "sensory perception of light stimulus" (GO:0050953), and "Phototransduction" (hsa04744). We further compared the functional enrichment results to two prior studies of glaucoma [6, 18]. As the results, six GO terms (in top 15) and 14 KEGG pathways (in top 20) were found mutual to both prior studies and this study, which affirmed that the selected module was the potential DIG-related gene module (Additional file 8: Figure S2).

### Identify glaucoma-associated genes

**Fig. 3:**
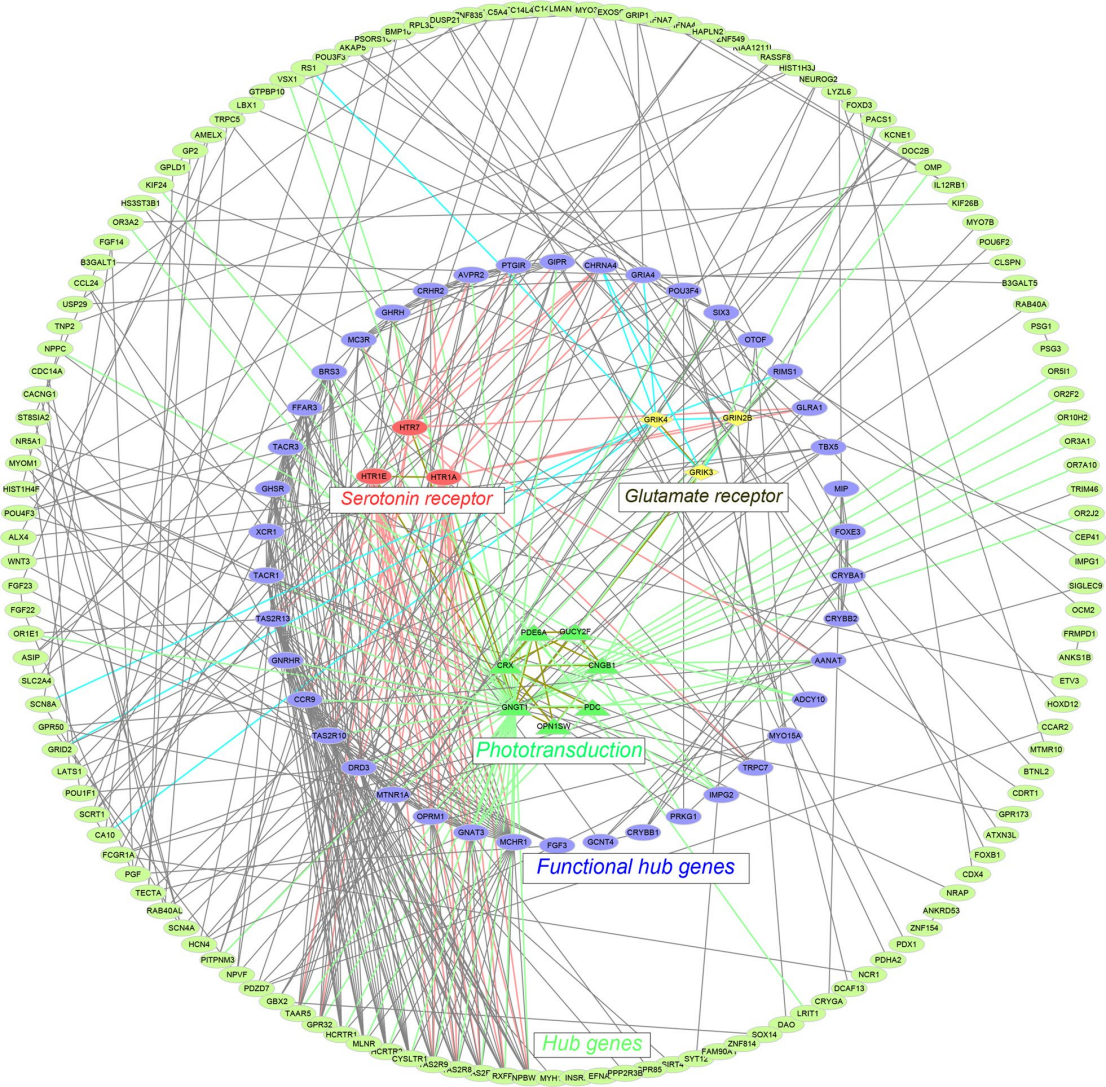
The glaucoma-related gene interaction network. This network was constructed in basis of 326 highly connected genes and corresponding 495 protein–protein interactions using the CytoScape tool. The purple nodes in the middle circle were 42 glaucoma-associated genes, and the innermost nodes were 13 DIG-associated genes supported by literature surveillance

**Fig. 4:**
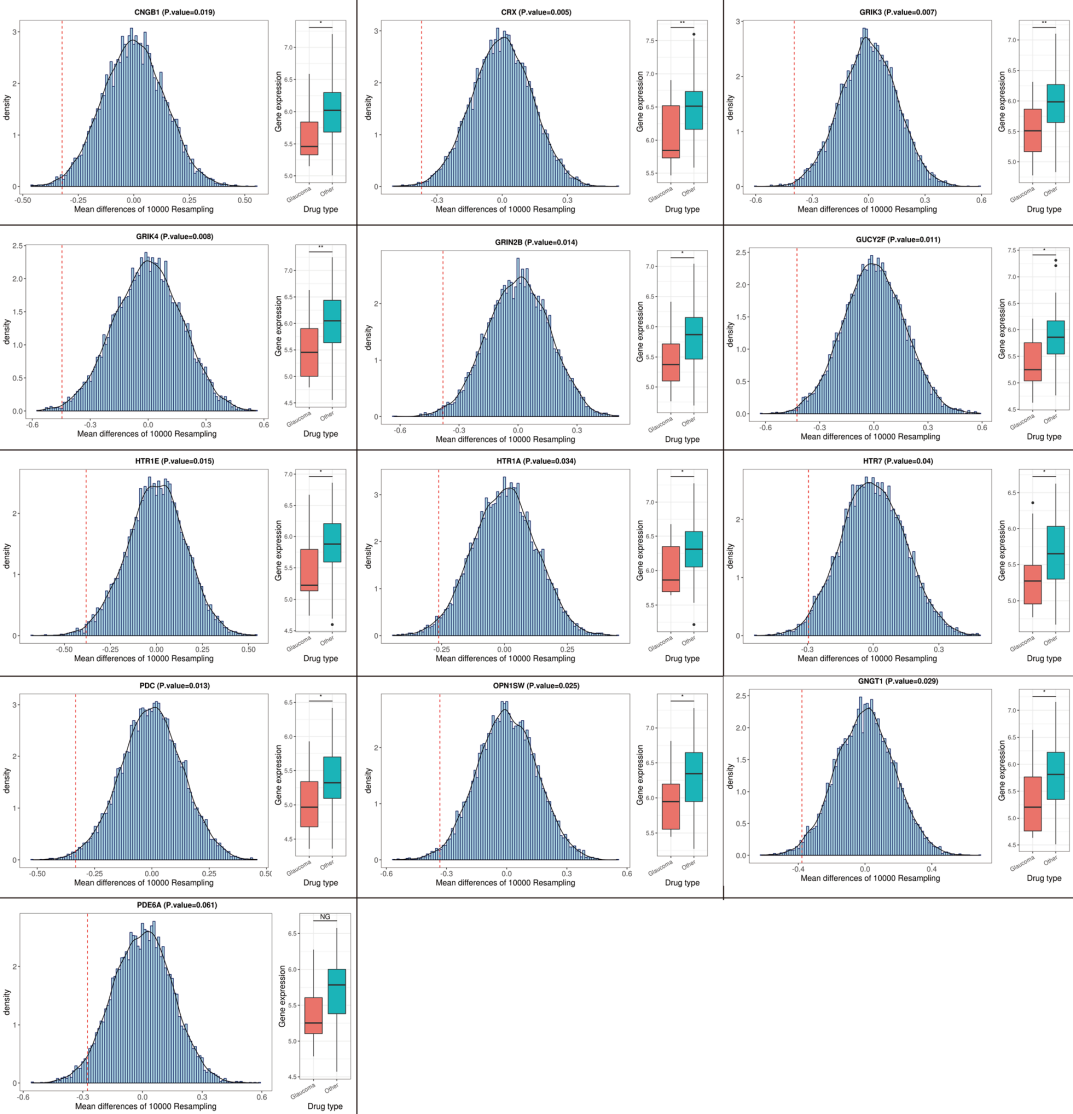
The permutation test of 13 glaucoma-associated genes. The test was conducted on 13 glaucoma-associated drugs and 63 non-glaucoma-inducing drugs. The density plot illustrated the distribution of gene expression difference after 10,000 resamplings. The red line stands for the position of real expression difference on the distribution curve. The boxplot illustrated the average gene expression changes under the treatment of two drug groups. The symbol * stands for the significance *p* value < 0.05 and ** stands for *p* value < 0.01

To identify potential DIG-associated genes/proteins in the DIG-related gene module, we carried out serial network analyses step by step. Firstly, we calculated the intra-module network connectivity for all genes in the DIG-related module. The calculation determined 326 highly connected genes by satisfying the criteria of connectivity > 0.95. These highly connected genes likely played centralized roles in the DIG-related gene interaction network. Subsequently, we searched these 326 genes against the STRING database, which identified 495 additional gene/protein interactions by meeting the criteria of the PPI enrichment significance *p* < 0.01. Upon the 326 genes and corresponding 495 interactions, we constructed a PPI network using the Cytoscape software. On basis of the PPI network, we further extracted a core subnetwork of the most enrichment (having the most connectivity) using the Cytoscape tool CytoHubba. This core subnetwork included 55 hub genes, which were most likely associated with DIG. Of them, 13 genes were previously reported to be associated with the sensory functions (Fig. 3). These 13 glaucoma-associated genes can be divided into three functional groups: phototransduction-related genes (*GNGT1*,* OPN1SW*,* PDE6A*, *PDC*, *CRX*, *CNGB1*, *GUCY2F*), glutamate receptor (GR) genes (*GRIN2B*, *GRIK3*, *GRIK4*), and 5-hydroxytryptamine receptor (HTR) genes (*HTR1A*, *HTR1E*, *HTR7*). Their connections with sensory functions and glaucoma were concluded in Table 1. It is a reasonable assumption that the 55 glaucoma-associated genes may react more actively to the treatment of the 13 glaucoma-associated drugs than that of the 63 non-glaucoma-inducing drugs. To prove the assumption, we carried out the permutation test. As the results, all of the 55 genes showed lower, 50 significantly (*p* < 0.05) and 5 insignificantly, gene expressions in response to the glaucoma-associated drugs. Furthermore, we observed a positive correlation (Pearson correlation coefficient = 0.56, *p* = 1.04e−5) between the differential significance (−log_10_*p_*value) and glaucoma-gene association strength (OR) of these 55 genes (Additional file 9: Figure S3). For the 13 reported glaucoma-associated genes, 12 genes had significantly (*p* < 0.05) lower expressions under the treatment of the glaucoma-associated drugs than that of non-glaucoma-associated drugs. The remaining one *PDE6A* showed a clearly lower expression but statistically insignificant (*p* = 0.061) (Fig. 4). All these results further supported that the down-regulation of the 55 genes likely answered for the DIGs.

**Table 1:** The information of 13 glaucoma-associated genes

Group	Gene	Tissue/cell specificity	Possible function in DIG
Phototransduction-related	CRX	Photoreceptor cells in the retina	Regulates photoreceptor-specific genes [19]
	PDC	Rod cells in the retina	Regulates the phototransduction cascades [20]
	CNGB1	Rod photoreceptor outer segment	Facilitates the regulation of ion flow [21]
	GUCY2F	Photoreceptor cells in the retina	Regulates the state transition of phototransduction [19]
	OPN1SW	Cone cells in the retina	Responds to normal color vision and activates phototransduction cascades [22]
	GNGT1	Retinal rod outer segment cells	Influences the formation and transmission of vision information [22]
	PDE6A	Retinal rod outer segment cells	Conducts and expands visual signals [23]
Glutamate receptor	GRIK3	Brain, eye, endocrine tissues	Mediates glutamate-mediated neural signals between photoreceptors and bipolar cells [24]
	GRIN2B		
	GRIK4		
5-Hydroxytryptamine receptor	HTR1A	Brain	Influences the intraocular pressure and the activity of RGCs [25]
	HTR1E		
	HTR7

The down-regulation of these genes by glaucoma-associated drugs were also reported in previous studies. For instance, dexamethasone and triamcinolone are commonly used in clinical practice to treat a wide range of retinal pathologies and both can induce glaucoma [26]. In mice, *OPN1SW, GRIN2B, HTR1A*, and *HTR7* were found downregulated after retina postintravitreal injections of triamcinolone [27]. *CNGB1*,* HTR1A*,* HTR7*,* OPN1SW*, and *CRX* were downregulated by dexamethasone in the retinal pigment epithelium of mouse [28].

### Discover potential biomarker genes for DIG

**Fig. 5:**
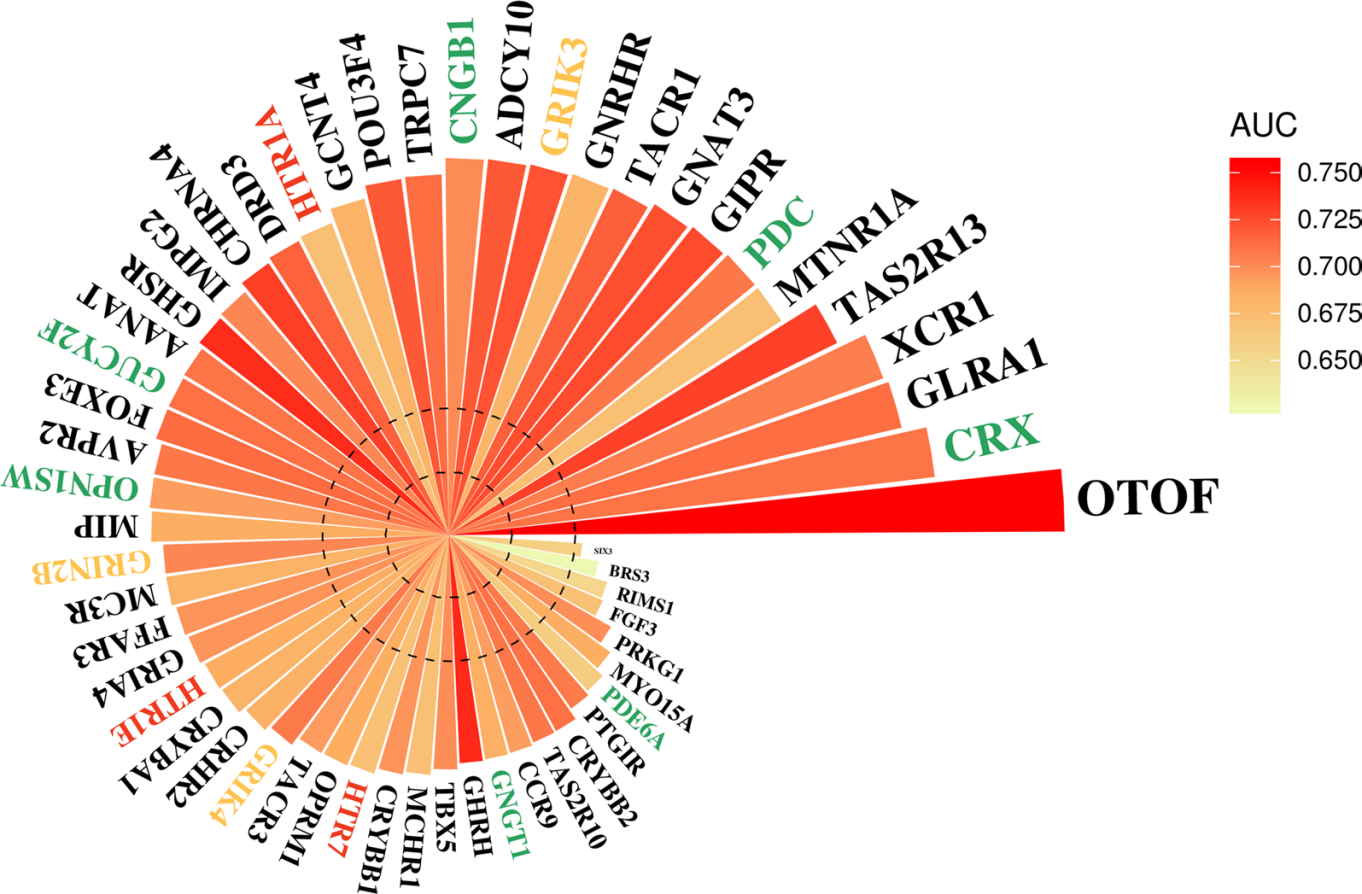
The pathogenic risk assessment of 55 glaucoma-associated genes. The bar length is positively proportional to the odds ratio (OR) value of the gene-to-glaucoma; the bar color is proportional to the AUC value for the gene ability in differentiating the glaucoma-associated drugs and non-glaucoma-inducing drugs. The color of gene symbol corresponds to different gene group: green stands for the phototransduction-related gene, yellow stands for the Glutamate receptor gene, red stands for the 5-hydroxytryptamine receptor gene, and black stands for the others

A good gene biomarker should be able to indicate disease incidence and as well be highly sensitive to the right drug treatment. The above analyses have confirmed the association of 55 genes with glaucoma. Here, we further quantitatively examined the pathogenic risk of these genes to glaucoma and their sensitivity to the glaucoma-associated drugs. For this purpose, we calculated the odds ratio (OR) for every 55 genes to glaucoma. And meanwhile, we determined the AUC for every gene alone in differentiating glaucoma-associated drugs and non-glaucoma-inducing drugs. As illustrated in Fig. 5 and Additional file 6: Table S6, 9 out of 55 genes exhibited highly risky to glaucoma and highly sensitive to glaucoma-associated drugs (OR > 6 and AUC > 0.7). These nine genes (*OTOF*, *CRX*, *GLRA1*, *XCR1*, *TAS2R13*, *PDC*, *GIPR*, *GNAT3*, and *TACR1*) could constitute a gene panel for diagnosing DIG. In particular, the otoferlin gene (*OTOF*) exhibited the riskiest to glaucoma with OR = 9.727, and it also showed remarkable ability in discriminating the glaucoma-inducing drugs from the non-glaucoma-inducing drugs with an AUC = 0.755. Hence, *OTOF* may serve as a potential biomarker gene for indicating DIGs.

## Discussion

The drug-induced glaucoma (DIG) has been encountered in normal chemotherapy for a long time but has not yet been fully understood. One of the major reasons is that glaucoma-induced drugs cannot provide suggestive chemical or protein target clues. In this study, we identified 13 strong glaucoma-associated drugs via mining 9,772,360 historical ADEs in the FAERS. With no surprise, these 13 drugs did not show significant structural or physicochemical similarity, which made it hard to explain the DIGs in a conventional way of drug-target interactions. Alternatively, we analyzed the mutual gene expression patterns treated by the 13 DIG-associated drugs using the WGCNA method, via which we uncovered a potential DIG-related gene module (network) and 55 potential glaucoma-associated genes. Subsequent network analyses and expression difference analysis further consolidated that the 55 genes might play central roles in the glaucoma development. Of these glaucoma-associated genes, 13 genes were literature-reported and can be divided into three functional gene groups according to their possible roles in glaucoma development, including seven phototransduction-related genes, three GR genes, and three HTR genes. According to the functional gene groups, we proposed here three possible routes, but not limited to, to induce glaucoma: phototransduction dysfunction, intracellular calcium homeostasis disruption, and retinal ganglion cell (RGC) death.

In clinical practices, glaucoma was always accompanied with several symptoms, such as elevated intraocular pressure (IOP), visual field defect, and optic nerve damage [1, 3]. Phototransduction is one of the main processes in vision formation. Of the seven phototransduction-related genes, *GNGT1* (the guanine nucleotide-binding protein G (T) subunit gamma-T1) is specifically expressed in the retinal rod outer segment cells, participating in the signal transduction process of cyclic GTP-specific phosphodiesterase as a modulator or transducer of light transduction. Down-regulation of *GNGT1* may result in blockage of light conduction, leading to visual field defect and vision loss. A previous study has shown that elimination of *GNGT1* could cause a dramatic decrease of the detected light signal in the intact mouse rods, a striking decline in the rod visual sensitivity, and eventually severe impairment of the nocturnal vision [29]. For other phototransduction-related genes *CNGB1, CRX, GUCY2F, OPN1SW*, *PDC*, and *PDE6A*, they may participate in the regulation of the state transition and the phototransduction cascade via interacting with *GNGT1* closely.

Glutamate receptors (GRs) and 5-hydroxytryptamine receptors (HTRs) are pivot proteins in maintaining the homeostasis of various neurotransmitters, the transmission of visual information, and the activity of nerve cells. A prior study found that abnormal increase of glutamate concentration in the vitreous bodies of humans and monkeys with glaucoma [30]. Over-activation of NMDA glutamate receptors (*GRIN2B*) affected the intracellular calcium homeostasis and induced cell apoptosis via caspases and PARP [31, 32], particularly the death of RGCs. The RGC was thought to be the most susceptible cell type to clinical glaucoma [33], and the RGC apoptosis was often observed in human and experimental animal models of glaucoma [34]. For the kainic acid (KA) glutamate receptors (*GRIK3* and *GRIK4*), they were acknowledged to mediate glutamate-mediated neural signals between photoreceptors and bipolar cells [24, 35]. Abnormal regulation of *GRIKs* may lead to the defect transmission of visual information. For the three HTRs (*HTR1A*, *HTR1E*, and *HTR7*), limited works have been undertaken to link them with glaucoma. However, prior work used *HTR2* (serotonin-2 receptor) agonists to reduce ocular hypotension in the chronic glaucoma model [36], which hint the HTRs might be involved in the IOP and glaucoma.

For the 55 glaucoma-associated genes, we further measured their pathogenic risks to glaucoma, as well as their sensitivity to the glaucoma-associated drugs. These intentions quantitatively ranked gene susceptibility to glaucoma, which finally suggested a nine-gene panel (*OTOF*, *CRX*, *GLRA1*, *XCR1*, *TAS2R13*, *PDC*, *GIPR*, *GNAT3*, and *TACR1*), which showed remarkable capability in differentiating glaucoma onset or not. *OTOF* encodes an integral membrane protein, which is implicated in a late stage of exocytosis and plays a ubiquitous role in synaptic vesicle trafficking. It was known to be a cause of neurosensory nonsyndromic recessive deafness via modulating γ-aminobutyric acid (GABA) activity, the metabolite of an excitatory neurotransmitter glutamate [37]. However, it is suspected that defective OTOF activity would markedly interfere GABA and glutamate metabolism [37], for example, causing abnormal accumulation of glutamate in the retina. The excessive glutamates would likely induce glutamate excitotoxicity to retinal neurons by overstimulation of glutamate receptors [38, 39]. Both the cone-rod homeobox (CRX) gene and the phosducin (PDC) gene are phototransduction-related genes. CRX is a photoreceptor-specific transcription factor that is necessary for the maintenance of normal cone and rod function. PDC encodes a phosphoprotein in the rod cells of retina, which may participate in the regulation of visual phototransduction or in the integration of photoreceptor metabolism. Several previous studies indicated that defects of CRX or PDC might answer for photoreceptor degeneration, leading to cone swelling and loss of photoreceptor cells, and eventually causing blindness [40–43]. The gastric inhibitory polypeptide receptor (*GIPR*) can stimulate insulin release in the presence of elevated glucose; variants of GIPR were found linked with diabetes-related primary OAG [44]. For the remaining five biomarker genes, no substantial evidences have been raised till now to link with glaucoma directly yet, which desires further experimental investigation.

This work has its limitations. First of all, the gene network study takes the advantage by bypassing the conventional challenge in discovering drug-target interactions beneath the DIGs; at the meanwhile, it ignores the diverse upstream mechanisms to trigger the DIGs, for instance, the genetic characteristics, the pathogenic conditions, and the drug types such as corticosteroids and anticholinergic drugs. Moreover, the results of this work could be limited by the data availability that some drugs were excluded in the WCGNA analysis due to the lack of sufficient drug-treated gene expression profiles. Nevertheless, this work provides a new insight into the systematic understanding of drug-induced glaucoma from a gene interaction perspective. The same strategy can be easily applied to mechanistically investigate other severe adverse drug reactions. It will also help prevent drug-induced glaucoma in clinical practice by suggesting the potential biomarkers for glaucoma prognosis.

## Conclusions

In summary, the development of DIG is a sophisticated process that involves multiple genes and pathways. Current molecular understanding of DIGs is mostly focused on sporadic genes or pathways, which show undetermined impacts on glaucoma. In this work, we develop a gene network analysis strategy to illustrate three possible molecular routes to induce glaucoma. These three routes are not isolated but connected in a glaucoma-related gene network. For the first time, we propose a list of genes highly susceptible to glaucoma. In particular, *OTOF* shows promising prognostic potential to drug-induced glaucoma in clinics.

## Supplementary Information

**Additional file 1**. Previously reported DIG-related genes.

**Additional file 2**. Detailed information of 1,114 gene expression profiles from the LINCS project.

**Additional file 3**. The mapping of the ATC code and cell line.

**Additional file 4**. The list of significant drug-glaucoma associations (OR > 2 & p < 0.05).

**Additional file 5**. The information of 13 glaucoma-associated drugs.

**Additional file 6**. The results of pathogenic risk assessment.

**Additional file 7**. The eigengene dendrogram constructed by the WGCNA method.

**Additional file 8**. The illustration of gene ontology enrichment.

**Additional file 9**. The correlation analysis between the differential significance and glaucoma-gene association strength.

## Data Availability

The drug-ADR relations were collected from the Adverse Drug Reaction Classification System (http://bio-add.org/ADReCS/download.jsp). The drug-treated gene expression profiles were downloaded from the GEO public database (https://www.ncbi.nlm.nih.gov/geo/query/acc.cgi?acc=GSE70138). The adverse drug event (ADE) reports were downloaded from openFDA (https://open.fda.gov/data/downloads/).

## Abbreviations

DIG: Drug-induced glaucoma
WGCNA: Weighted gene co-expression network analysis
OAG: Open-angle glaucoma
IOP: Intraocular pressure
CAG: Closed-angle glaucoma
FAERS: FDA Adverse Event Reporting System
GWAS: Genome-Wide Association Studies
CD: Characteristic Direction
DEG: Differentially expressed gene
ADE: Adverse drug event
OR: Odds ratio
ME: Module eigengene
MM: Module membership
PPI: Protein–protein interaction
GO: Gene Ontology
FDR: False discovery rate
ROC: Curve receiver operating characteristic curve
AUC: Area under the curve
CV: Coefficient of variation
HTR: 5-Hydroxytryptamine receptor
RGC: Retinal ganglion cell
GR: Glutamate receptor
