# Supplementary material for: Gene network analyses unveil possible molecular basis underlying drug-induced glaucoma
Source: BMC Med Genomics. 2021 Apr 19;14:109. doi: 10.1186/s12920-021-00960-9 (PMC8056654; doi:10.1186/s12920-021-00960-9)
Supplement: Supplementary file 1 — Additional file 1. Previously reported DIG-related genes. [file 12920_2021_960_MOESM1_ESM.docx]

**Table S1. Previously reported DIG-related genes.**

| **Drug classification** | **Drug name** | **DIG-related gene** | **Correlation with DIG** | **Reference (PMID)** |
| --- | --- | --- | --- | --- |
| Steroid | Dexamethasone | MYOC | MYOC can cause an increase in IOP through obstruction of the aqueous outflow. | 30816137, 29499052 |
|  | Prednisolone | GPNMB | GPNMB can interact with STC1, then cause neuronal degeneration and RGC death, resulting in elevated IOP. | 21980000, 31057322 |
|  | Triamcinolone | HCG22 | Secretion of HCG22 protein in the aqueous outflow pathways is related with IOP. | 25813999 |
| Anticholinergics | Diazepam | TSPO | TSPO expression in the retina was primarily assigned to reactive microglia. | 29211020, 30777091 |
