# Supplementary material for: Gene network analyses unveil possible molecular basis underlying drug-induced glaucoma
Source: BMC Med Genomics. 2021 Apr 19;14:109. doi: 10.1186/s12920-021-00960-9 (PMC8056654; doi:10.1186/s12920-021-00960-9)
Supplement: Supplementary file 3 — Additional file 3. The mapping of the ATC code and cell line. [file 12920_2021_960_MOESM3_ESM.docx]

**Table S3. The mapping of the ATC code and Cell line**

| **System Organ Class (ATC code)** | **Cell line (Tissue)** |
| --- | --- |
| Alimentary tract and metabolism (A) | HT29 (Large intestine)  PHH (Liver) |
| Dermatologicals (D) | A375 (Skin) |
| Genitourinary system and sex hormones (G) | HA1E (Kidney)  PC3 (Prostate)  VCAP (Prostate)  MCF7 (Breast) |
| Respiratory system (R) | A549 (Lung)  HCC515 (Lung) |
