# Supplementary material for: Gene network analyses unveil possible molecular basis underlying drug-induced glaucoma
Source: BMC Med Genomics. 2021 Apr 19;14:109. doi: 10.1186/s12920-021-00960-9 (PMC8056654; doi:10.1186/s12920-021-00960-9)
Supplement: Supplementary file 4 — Additional file 4. The list of significant drug-glaucoma associations (OR > 2 & p < 0.05). [file 12920_2021_960_MOESM4_ESM.docx]

**Table S4. The list of significant drug-glaucoma associations (OR>2 & *p*<0.05)**

| **DRUG_NAME** | **ATC** | **OR** | ***p* value** |
| --- | --- | --- | --- |
| Imipramine | N06AA02 | 175.15 | 6.40E-5 |
| Vardenafil | G04BE09 | 99.91 | 2.12E-4 |
| Topiramate | N03AX11 | 89.85 | 0 |
| Prednisolone | A07EA01; C05AA04; D07AA03; D07XA02; H02AB06; R01AD02; S01BA04; S01CB02; S02BA03; S03BA02 | 47.36 | 1.29E-11 |
| Ipratropium | R01AX03; R03BB01 | 29.90 | 4.03E-11 |
| Betamethasone | A07EA04; C05AA05; D07AC01; D07XC01; H02AB01; R01AD06; R03BA04; S01BA06; S01CB04; S02BA07; S03BA03 | 26.13 | 2.00E-5 |
| Ropinirole | N04BC04 | 18.22 | 6.64E-4 |
| Zonisamide | N03AX15 | 16.10 | 7.14E-3 |
| Hydrocortisone | A01AC03; A07EA02; C05AA01; D07AA02; D07XA01; H02AB09; S01BA02; S01CB03; S02BA01 | 15.78 | 7.38E-3 |
| Dexamethasone | A01AC02; C05AA09; D07AB19; D07XB05; D10AA03; H02AB02; R01AD03; S01BA01; S01CB01; S02BA06; S03BA01 | 13.27 | 4.01E-16 |
| Brimonidine | D11AX21; S01EA05 | 12.84 | 1.78E-3 |
| Escitalopram | N06AB10 | 11.70 | 6.66E-24 |
| Mometasone | D07AC13; D07XC03; R01AD09; R03BA07 | 10.91 | 2.89E-15 |
| Citalopram | N06AB04 | 6.64 | 4.89E-13 |
| Methylphenidate | N06BA04 | 5.57 | 2.98E-1 |
| Fluoxetine | N06AB03 | 5.30 | 2.41E-8 |
| Prednisone | A07EA03; H02AB07 | 4.75 | 1.62E-4 |
| Desvenlafaxine | N06AX23 | 4.71 | 7.66E-5 |
| Budesonide | A07EA06; D07AC09; R01AD05; R03BA02 | 4.34 | 3.32E-2 |
| Pregabalin | N03AX16 | 2.73 | 8.79E-6 |
| Sildenafil | G04BE03 | 2.65 | 2.60E-3 |
| Quetiapine | N05AH04 | 2.59 | 1.20E-4 |
| Duloxetine | N06AX21 | 2.41 | 1.07E-3 |
| Venlafaxine | N06AX16 | 2.34 | 1.73E-2 |
| Olanzapine | N05AH03 | 2.23 | 1.40E-2 |
