# Supplementary material for: Gene network analyses unveil possible molecular basis underlying drug-induced glaucoma
Source: BMC Med Genomics. 2021 Apr 19;14:109. doi: 10.1186/s12920-021-00960-9 (PMC8056654; doi:10.1186/s12920-021-00960-9)
Supplement: Supplementary file 5 — Additional file 5. The information of 13 glaucoma-associated drugs. [file 12920_2021_960_MOESM5_ESM.docx]

**Table S5. The information of 13 glaucoma-associated drugs**

| **Drug name** | **ADReCS ID** | **L1000 ID** | **ATC** | **OR** | ***p* value** |
| --- | --- | --- | --- | --- | --- |
| Homatropine | BADD_D01075 | BRD-A09539288 | S |  |  |
| Scopolamine | BADD_D01994 | BRD-A62035778 | A;N;;S |  |  |
| Atropine | BADD_D00189 | BRD-M35773784 | A;S |  |  |
| Cortisone | BADD_D00532 | BRD-K86161929 | [H;S](https://www.whocc.no/atc_ddd_index/?code=S01BA03) |  |  |
| Dexamethasone | BADD_D00623 | BRD-A69951442 | [A;C;D;H;R;S](https://www.whocc.no/atc_ddd_index/?code=S03BA03) | 13.27 | 4.00e-16 |
| Medrysone | BADD_D01367 | BRD-K56515112 | S |  |  |
| Betamethasone | BADD_D00260 | BRD-A02180903 | [A;C;D;H;R;S](https://www.whocc.no/atc_ddd_index/?code=S03BA03) | 26.12 | 2.00e-5 |
| Fluorometholone | BADD_D00929 | BRD-A13133631 | C;D;S |  |  |
| Hydrocortisone | BADD_D01086 | BRD-A75172220 | A;C;D;H;S | 15.78 | 7.38e-3 |
| Prednisolone | BADD_D01831 | BRD-A27887842 | [A;C;D;H;R;S](https://www.whocc.no/atc_ddd_index/?code=S03BA03) | 47.36 | 1.29e-11 |
| Brimonidine | BADD_D00294 | BRD-K68264559 | D;S | 12.84 | 1.78e-3 |
| Ganciclovir | BADD_D01003 | BRD-K22662435 | J;S |  |  |
| Triamcinolone | BADD_D02271 | BRD-A37780065 | [A;C;D;H;R;S](https://www.whocc.no/atc_ddd_index/?code=S03BA03) | 4.50 | 7.41e-2 |
