# Supplementary material for: Gene network analyses unveil possible molecular basis underlying drug-induced glaucoma
Source: BMC Med Genomics. 2021 Apr 19;14:109. doi: 10.1186/s12920-021-00960-9 (PMC8056654; doi:10.1186/s12920-021-00960-9)
Supplement: Supplementary file 6 — Additional file 6. The results of pathogenic risk assessment. [file 12920_2021_960_MOESM6_ESM.docx]

**Table S6. The results of pathogenic risk assessment.**

| **Gene name** | **Gene group** | **Module membership** | **OR** | ***p* value** | **AUC** |
| --- | --- | --- | --- | --- | --- |
| **OTOF** | Other | 0.969 | 9.727 | 0.002 | 0.755 |
| **CRX** | Phototransduction-related | 0.954 | 7.73 | 0.006 | 0.709 |
| **GLRA1** | Other | 0.974 | 7.349 | 0.011 | 0.712 |
| **XCR1** | Other | 0.973 | 7.293 | 0.014 | 0.706 |
| **TAS2R13** | Other | 0.985 | 6.841 | 0.004 | 0.729 |
| MTNR1A | Other | 0.977 | 6.245 | 0.024 | 0.673 |
| **PDC** | Phototransduction-related | 0.951 | 6.241 | 0.011 | 0.708 |
| **GIPR** | Other | 0.952 | 6.205 | 0.008 | 0.725 |
| **GNAT3** | Other | 0.963 | 6.152 | 0.006 | 0.724 |
| **TACR1** | Other | 0.976 | 6.053 | 0.008 | 0.718 |
| GNRHR | Other | 0.96 | 6.025 | 0.023 | 0.681 |
| GRIK3 | Glutamate receptor | 0.953 | 5.99 | 0.007 | 0.723 |
| ADCY10 | Other | 0.971 | 5.963 | 0.011 | 0.72 |
| CNGB1 | Phototransduction-related | 0.982 | 5.95 | 0.018 | 0.701 |
| TRPC7 | Other | 0.976 | 5.691 | 0.026 | 0.712 |
| POU3F4 | Other | 0.971 | 5.668 | 0.011 | 0.72 |
| GCNT4 | Other | 0.961 | 5.473 | 0.029 | 0.683 |
| HTR1A | 5-hydroxytryptamine receptors | 0.978 | 5.258 | 0.034 | 0.672 |
| DRD3 | Other | 0.965 | 5.209 | 0.014 | 0.717 |
| CHRNA4 | Other | 0.981 | 5.147 | 0.005 | 0.73 |
| IMPG2 | Other | 0.976 | 5.006 | 0.006 | 0.702 |
| GHSR | Other | 0.958 | 4.971 | 0.002 | 0.737 |
| AANAT | Other | 0.968 | 4.881 | 0.009 | 0.709 |
| GUCY2F | Phototransduction-related | 0.964 | 4.865 | 0.012 | 0.711 |
| FOXE3 | Other | 0.983 | 4.864 | 0.028 | 0.713 |
| AVPR2 | Other | 0.976 | 4.749 | 0.013 | 0.708 |
| OPN1SW | Phototransduction-related | 0.974 | 4.745 | 0.022 | 0.694 |
| MIP | Other | 0.955 | 4.702 | 0.03 | 0.686 |
| GRIN2B | Glutamate receptor | 0.984 | 4.514 | 0.016 | 0.702 |
| MC3R | Other | 0.978 | 4.513 | 0.015 | 0.683 |
| FFAR3 | Other | 0.968 | 4.453 | 0.01 | 0.697 |
| GRIA4 | Other | 0.97 | 4.416 | 0.029 | 0.697 |
| HTR1E | 5-hydroxytryptamine receptors | 0.981 | 4.357 | 0.013 | 0.687 |
| CRYBA1 | Other | 0.975 | 4.337 | 0.028 | 0.683 |
| CRHR2 | Other | 0.962 | 4.234 | 0.045 | 0.684 |
| GRIK4 | Glutamate receptor | 0.983 | 4.14 | 0.011 | 0.707 |
| TACR3 | Other | 0.977 | 3.99 | 0.025 | 0.695 |
| OPRM1 | Other | 0.951 | 3.98 | 0.042 | 0.687 |
| HTR7 | 5-hydroxytryptamine receptors | 0.953 | 3.951 | 0.047 | 0.67 |
| CRYBB1 | Other | 0.961 | 3.855 | 0.006 | 0.697 |
| MCHR1 | Other | 0.956 | 3.804 | 0.031 | 0.673 |
| TBX5 | Other | 0.982 | 3.707 | 0.029 | 0.7 |
| GHRH | Other | 0.966 | 3.61 | 0.004 | 0.739 |
| GNGT1 | Phototransduction-related | 0.966 | 3.591 | 0.029 | 0.685 |
| CCR9 | Other | 0.96 | 3.578 | 0.008 | 0.694 |
| TAS2R10 | Other | 0.956 | 3.54 | 0.005 | 0.708 |
| CRYBB2 | Other | 0.955 | 3.531 | 0.012 | 0.711 |
| PTGIR | Other | 0.953 | 3.356 | 0.015 | 0.707 |
| PDE6A | Phototransduction-related | 0.958 | 3.299 | 0.055 | 0.662 |
| MYO15A | Other | 0.967 | 3.154 | 0.043 | 0.686 |
| PRKG1 | Other | 0.964 | 2.943 | 0.015 | 0.7 |
| FGF3 | Other | 0.968 | 2.644 | 0.06 | 0.67 |
| RIMS1 | Other | 0.972 | 2.61 | 0.074 | 0.653 |
| BRS3 | Other | 0.961 | 2.4 | 0.199 | 0.625 |
| SIX3 | Other | 0.974 | 2.111 | 0.062 | 0.659 |
