# Supplementary figures and images for: Gene network analyses unveil possible molecular basis underlying drug-induced glaucoma
Source: BMC Med Genomics. 2021 Apr 19;14:109. doi: 10.1186/s12920-021-00960-9 (PMC8056654; doi:10.1186/s12920-021-00960-9)

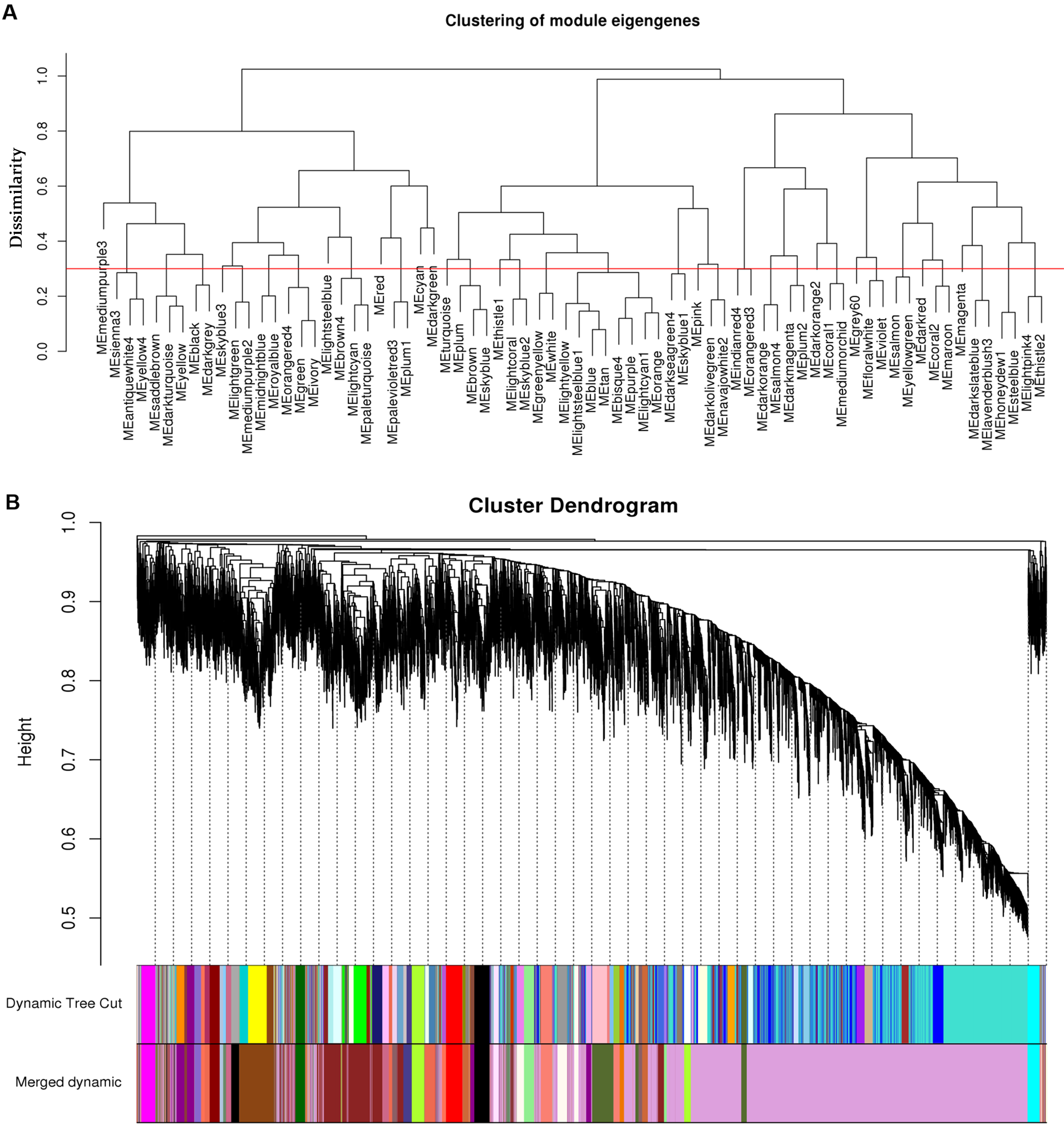

Supplement: Supplementary file 7 — Additional file 7. The eigengene dendrogram constructed by the WGCNA method. [file 12920_2021_960_MOESM7_ESM.tif]

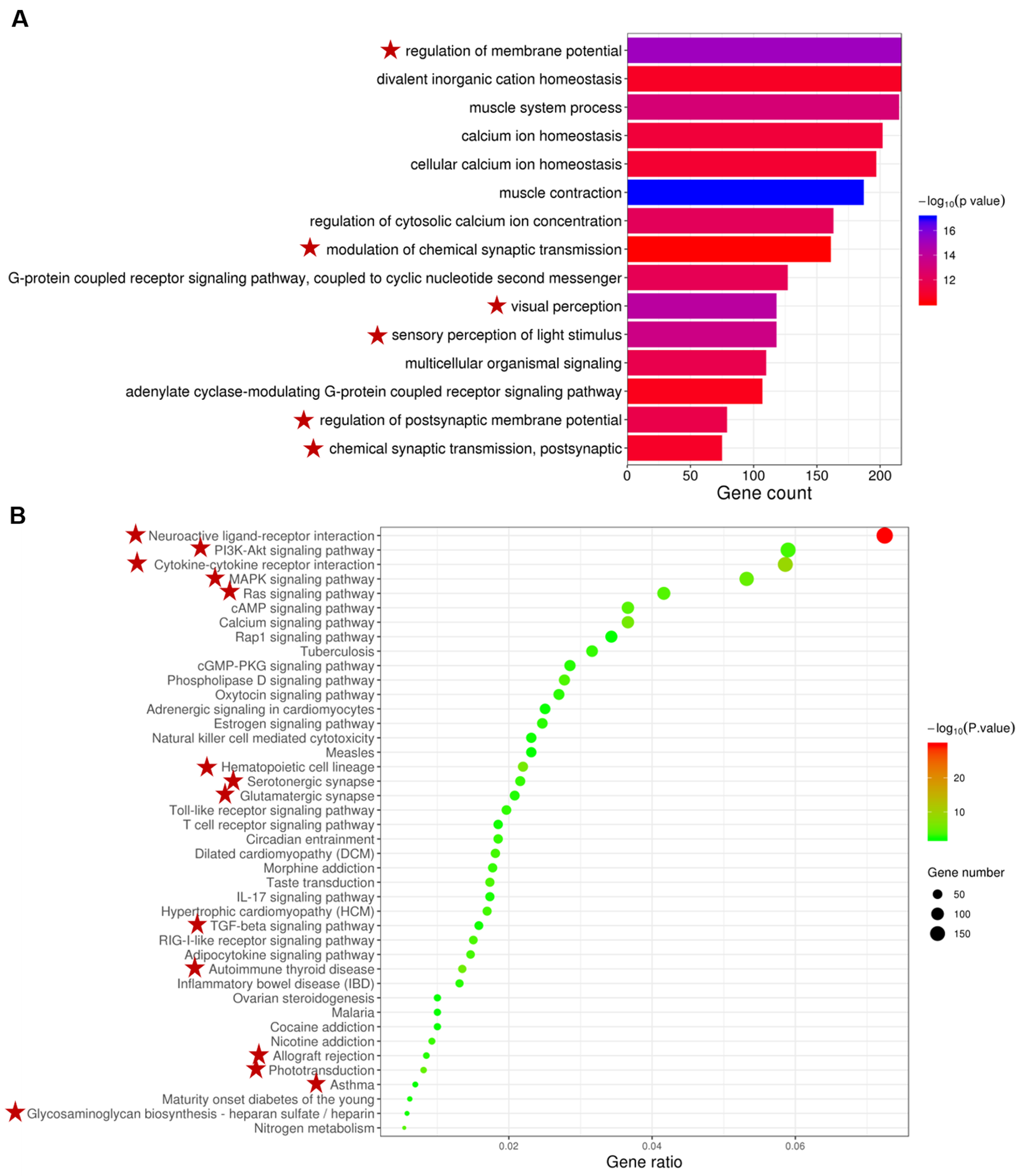

Supplement: Supplementary file 8 — Additional file 8. The illustration of gene ontology enrichment. [file 12920_2021_960_MOESM8_ESM.tif]

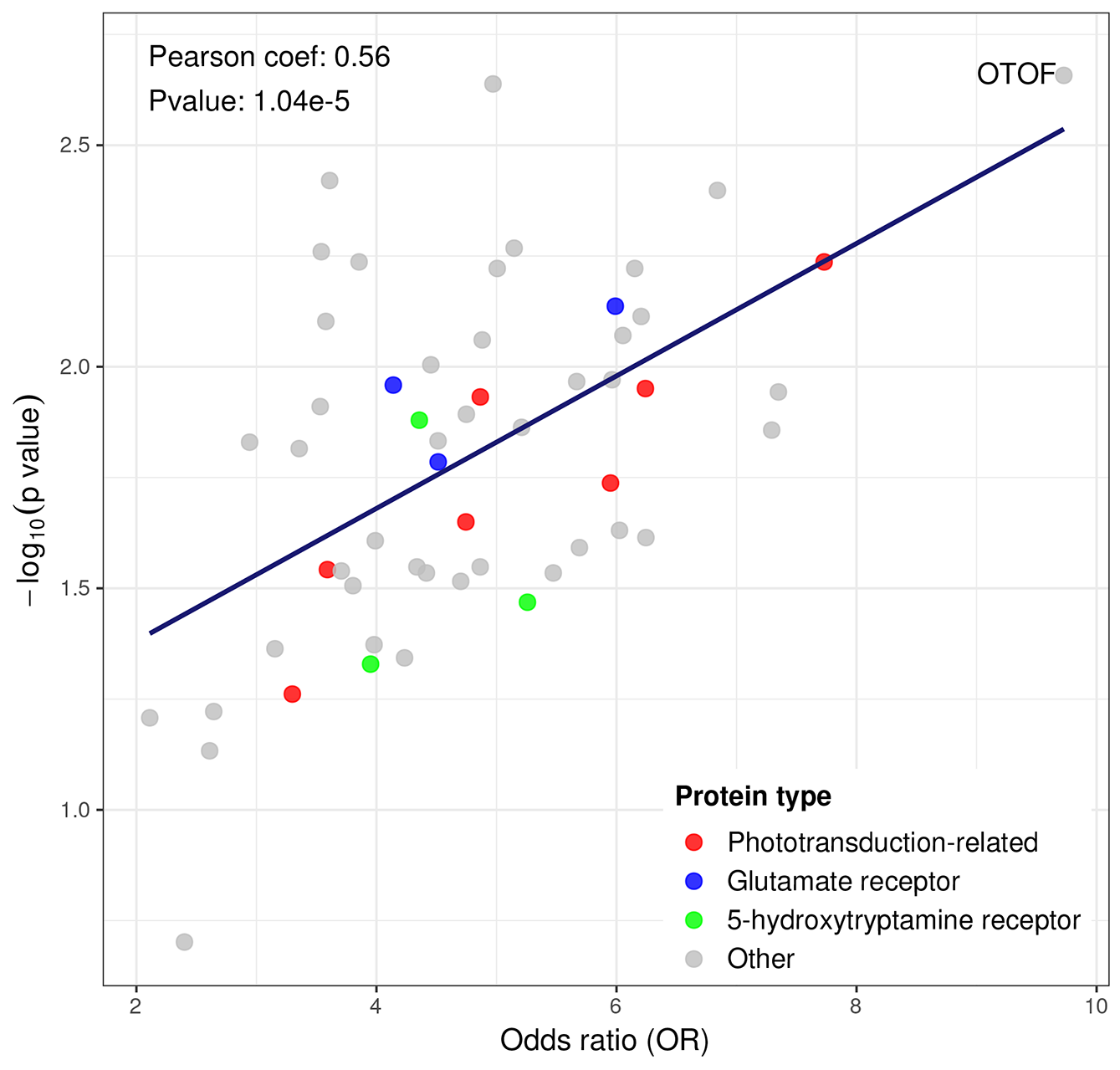

Supplement: Supplementary file 9 — Additional file 9. The correlation analysis between the differential significance and glaucoma-gene association strength. [file 12920_2021_960_MOESM9_ESM.tif]
